# The establishment of a multiple myeloma clinical registry in the Asia–Pacific region: The Asia–Pacific Myeloma and Related Diseases Registry (APAC MRDR)

**DOI:** 10.1186/s12874-024-02227-0

**Published:** 2024-05-02

**Authors:** Naomi Aoki, Pin-Yen Chen, Wenming Chen, Wee Joo Chng, Gin Gin Gan, Yeow Tee Goh, Jian Hou, Jeffrey Huang, Kihyun Kim, Je Jung Lee, Jin Lu, Zoe K. McQuilten, Chang Ki Min, Elizabeth Moore, Laura Oliver, Neil A. Waters, Cameron Wellard, Erica M. Wood, Su-Peng Yeh, Andrew Spencer, the APAC MRDR Investigators

**Affiliations:** 1 School of Public Health and Preventive Medicine, Monash University, Melbourne, Australia; 2 Beijing Chaoyang Hospital, Beijing, China; 3 National University Cancer Institute, Singapore, Singapore; 4 Universiti Malaya Medical Centre, Selangor, Malaysia; 5 Singapore General Hospital, Singapore, Singapore; 6 Renji Hospital, Shanghai, China; 7 National Taiwan University Hospital, Taipei City, Taiwan; 8 Sungkyunkwan University School of Medicine, Samsung Medical Center, Seoul, Korea; 9 Chonnam National University Hospital, Gwangju, Korea; 10 Peking University People’s Hospital, Beijing, China; 11 Monash Health, Melbourne, Australia; 12 Seoul St Mary’s Hospital, Seoul, Korea; 13 China Medical University Hospital, Taichung City, Taiwan; 14 The Alfred Hospital, Melbourne, Australia; 15 Asia-Pacific Myeloma and Related Diseases Registry, Public Health and Preventive Medicine, Monash University, 553 St Kilda Road, Melbourne, VIC 3004 Australia; 16 School of Translational Medicine, Monash University, Melbourne, Australia

**Keywords:** Multiple myeloma, Clinical registry, Epidemiology, Real-world data

## Abstract

**Background:**

Multiple myeloma (MM) is the second most common haematological cancer worldwide. Along with related diseases including monoclonal gammopathy of undetermined significance (MGUS), plasma cell leukaemia (PCL) and plasmacytoma, MM incidence is rising, yet it remains incurable and represents a significant disease burden. Clinical registries can provide important information on management and outcomes, and are vital platforms for clinical trials and other research. The Asia–Pacific Myeloma and Related Diseases Registry (APAC MRDR) was developed to monitor and explore variation in epidemiology, treatment regimens and their impact on clinical outcomes across this region. Here we describe the registry’s design and development, initial data, progress and future plans.

**Methods:**

The APAC MRDR was established in 2018 as a multicentre collaboration across the Asia–Pacific, collecting prospective data on patients newly diagnosed with MM, MGUS, PCL and plasmacytoma in Korea, Singapore, Malaysia and Taiwan, with China recently joining. Development of the registry required a multidisciplinary team of clinicians, researchers, legal and information technology support, and financial resources, as well as local clinical context from key opinion leaders in the APAC region. Written informed consent is obtained and data are routinely collected throughout treatment by hospital staff. Data are stored securely, meeting all local privacy and ethics requirements. Data were collected from October 2018 to March 2024.

**Results:**

Over 1700 patients from 24 hospitals have been enrolled onto the APAC MRDR to date, with the majority (86%) being newly diagnosed with MM. Bortezomib with an immunomodulatory drug was most frequently used in first-line MM therapy, and lenalidomide-based therapy was most common in second-line. Establishment and implementation challenges include regulatory and a range of operational issues.

**Conclusion:**

The APAC MRDR is providing ‘real-world’ data to participating sites, clinicians and policy-makers to explore factors influencing outcomes and survival, and to support high quality studies. It is already a valuable resource that will continue to grow and support research and clinical collaboration in MM and related diseases across the APAC region.

## Background

Multiple myeloma (MM) is the second most common haematological cancer worldwide. Despite advances in treatment that have extended survival, MM remains incurable, which has led to a greater prevalence and burden of disease [1–3]. Moreover, the incidence of MM is rising globally (by 126% from 1990–2016), with the greatest increase in Asia, specifically in China, Korea, and Taiwan (262%, 1990–2016) [1].

Therapeutic options for MM continue to expand with new targeted agents entering the therapeutic arena, but with little real-world evidence (RWE) on optimal use and outcomes of available therapies outside the limited scope of clinical trials. The discordance between outcomes in the real world and in pivotal MM randomised controlled trials informing drug approvals was highlighted in two studies, revealing significant bias toward inclusion of younger, fitter MM patients in clinical trials, particularly in relapsed MM [4, 5]. A further study showed that 80% of real-world relapsed MM patients would have been ineligible for one or more of four hallmark randomised controlled trials exploring the addition of new drugs to standard of care [6]. Apart from reflecting the experience of a broader, more diverse and representative MM population, RWE data is often captured over extended periods allowing assessment of long-term outcomes beyond limited clinical trial duration. In addition, RWE reflects the complexities of clinical practice, making results more generalisable; and by leveraging existing data sources such as registries, it is a more cost-effective means of generating evidence. RWE therefore complements clinical trial data, allowing a more comprehensive understanding of treatment performance in different contexts and settings.

Variation in access and use of novel treatments in routine clinical care are observed around the world and across the Asia–Pacific (APAC) region. While a full discussion of this variation is outside the scope of this manuscript, differences in local regulations, infrastructure, funding, and clinical practice all play a role and affect the uptake of new therapies and outcomes for patients [7, 8]. In this context, generation of RWE, including long-term monitoring and evaluation of current and future treatment strategies, is imperative to help inform optimal use of available MM therapies.

### Clinical registries

Registries permit standardised, long-term data collection and enable monitoring and benchmarking to improve clinical outcomes, quality of life, and cost-effectiveness of care for patients [9–12]. Multiple hospitals can be engaged across regions, to generate large, collated datasets, making registries particularly relevant for less common cancers, such as MM, where such data are limited. Numerous country-specific or ‘dual country’ MM registries or longitudinal observation studies have been set up including in Europe, Australia and New Zealand (ANZ), and the USA-based Connect MM® registry [13–19]. Amongst the main aims of many of these registries are to identify areas where improvements are needed, inform decision-making, support research, and enable benchmarking to promote best practice and consistent quality in MM care.

Other registries have recruited across multiple countries or regions with specific aims such as INSIGHT MM (USA, South and Central America, Europe, Middle East, East Asia: describes patterns in MM patients, treatment and outcomes), CoMMpass (USA, Canada, Spain, Italy: genomic/ clinical/ quality of life [QoL] MM dataset on treatment and outcomes), EMMOS (Europe, Israel, Algeria, South Africa: describes treatment/ progression/ QoL/ health economics by line of therapy), and PREAMBLE (USA, Canada, Western Europe: assesses clinical effectiveness of therapies) [20–23]; however the Asia–Pacific region, was scarcely represented in any of these projects.

The APAC Myeloma and Related Diseases Registry (APAC MRDR) was established in 2018 as a regional collaboration and sister registry to ANZ MRDR, to address an unmet need (ACTRN12618000659202) [13]. The aim is to collect and analyse a standardised dataset used to monitor treatment and response to therapy, to benchmark outcomes in patients with MM in participating APAC regions, and to explore variation in practice and access to care.

Here we provide a comprehensive description of the design and development of the APAC MRDR, an overview of progress to date, first-results and future plans.

## Methods

### Study design and participants

The APAC MRDR is a non-interventional, prospective, multicentre, clinical registry of adults newly diagnosed with MM, smouldering MM, plasma cell leukaemia, plasmacytoma, or monoclonal gammopathy of undetermined significance (MGUS) presenting to participating tertiary-care hospitals in the APAC region. This paper focuses on MM, which is the diagnosis of most registry patients (86%).

**Fig. 1:**
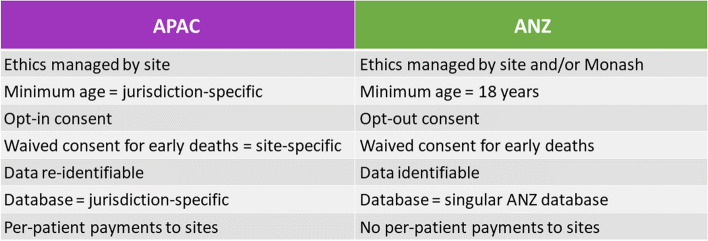
Features and differences of the APAC and ANZ MRDR

The registry design is based on the ANZ MRDR [10] to permit analyses across the two registries. Key differences between the two registries are summarised in Fig. 1. Adults with a new diagnosis are prospectively identified by treating physicians and considered eligible if they meet the International Myeloma Working Group criteria for myeloma or a related disease [24], and are diagnosed no earlier than three months prior to the Human Research Ethics Committee (HREC) or Institutional Review Board (IRB) approval for the site, to ensure the prospective nature of the registry.

### Setting

The APAC MRDR was first established as a pilot study in 2018 with five metropolitan hospitals from Korea and Singapore. Interested hospitals were invited to participate by the APAC MRDR Study Team or members of the Steering Committee through clinical networks. Region-specific customisation and implementation of the APAC MRDR databases, as well as operational and governance processes, were developed with input from the pilot hospitals and staff. Once operational, processes were confirmed and the databases for Korea and Singapore were created and tested, and additional hospitals and locations were invited to join the registry.

The APAC MRDR is established and recruiting in Korea and Singapore (since 2018), Malaysia (since 2019) and Taiwan (since 2021). China began patient recruitment in early 2024. Most participating hospitals are tertiary (academic) centres, and three private hospitals have joined the registry, with one further centre awaiting governance approval. Additional hospitals in the APAC region have been invited to participate and are anticipated to join.

### Ethics approval and consent to participate

Participating hospitals received HREC or IRB approvals (or equivalent governance approvals) before data collection commenced. Privacy legislation in participating APAC jurisdictions at the time of establishing the registry did not permit an ‘opt-out’ approach to consent, which is approved and used in the ANZ MRDR [10]; therefore, APAC MRDR participants, or their next of kin, must provide written informed consent before data collection commences. A patient’s legally acceptable representative can sign the consent on the patient’s behalf, if appropriate. Written informed consent has been obtained for all patients enrolled onto the APAC MRDR.

### Data collection

**Fig. 2:**
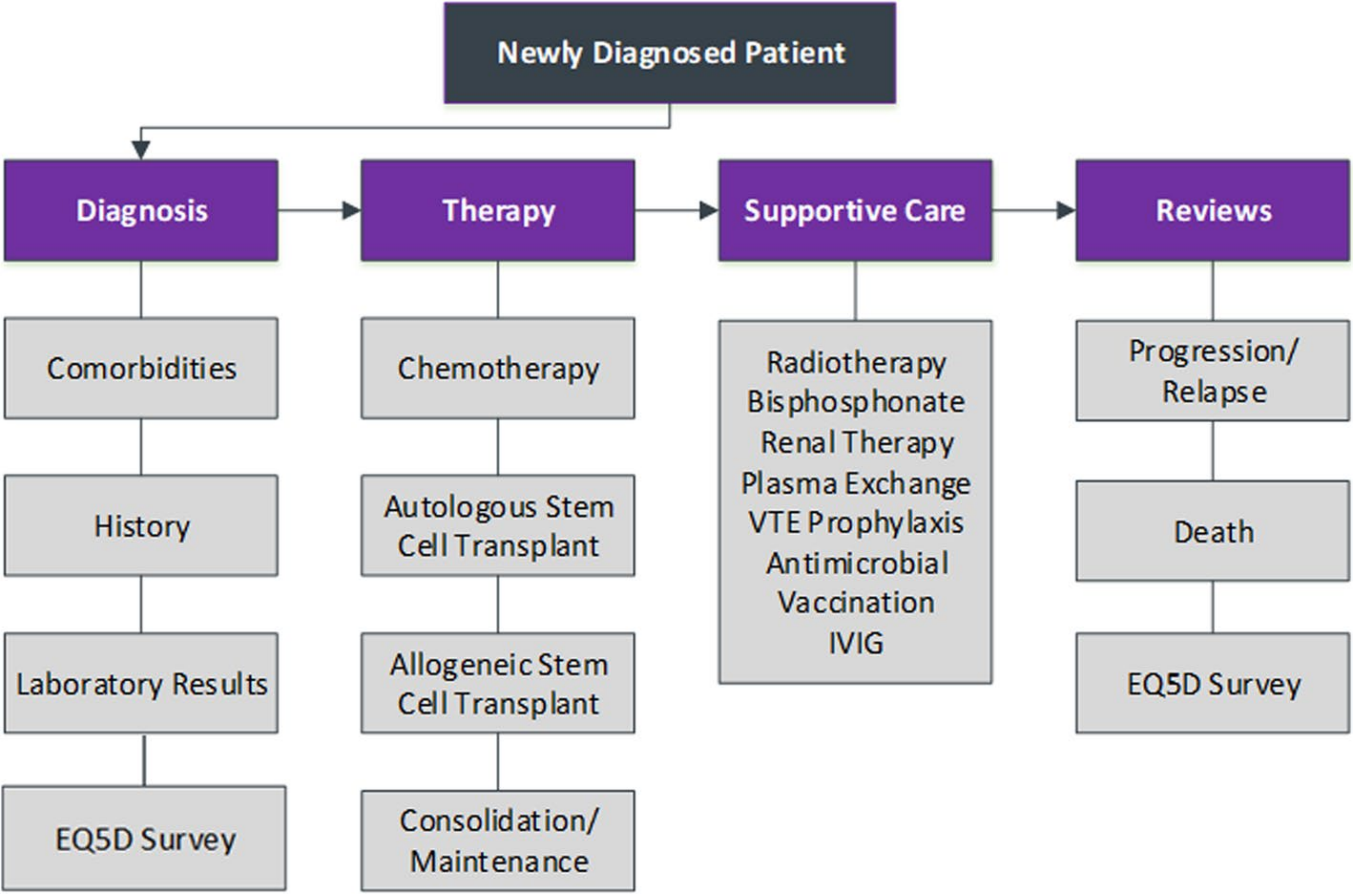
Data collection overview VTE: venous thromboembolism, IVIG: intravenous immunoglobulin

**Fig. 3:**
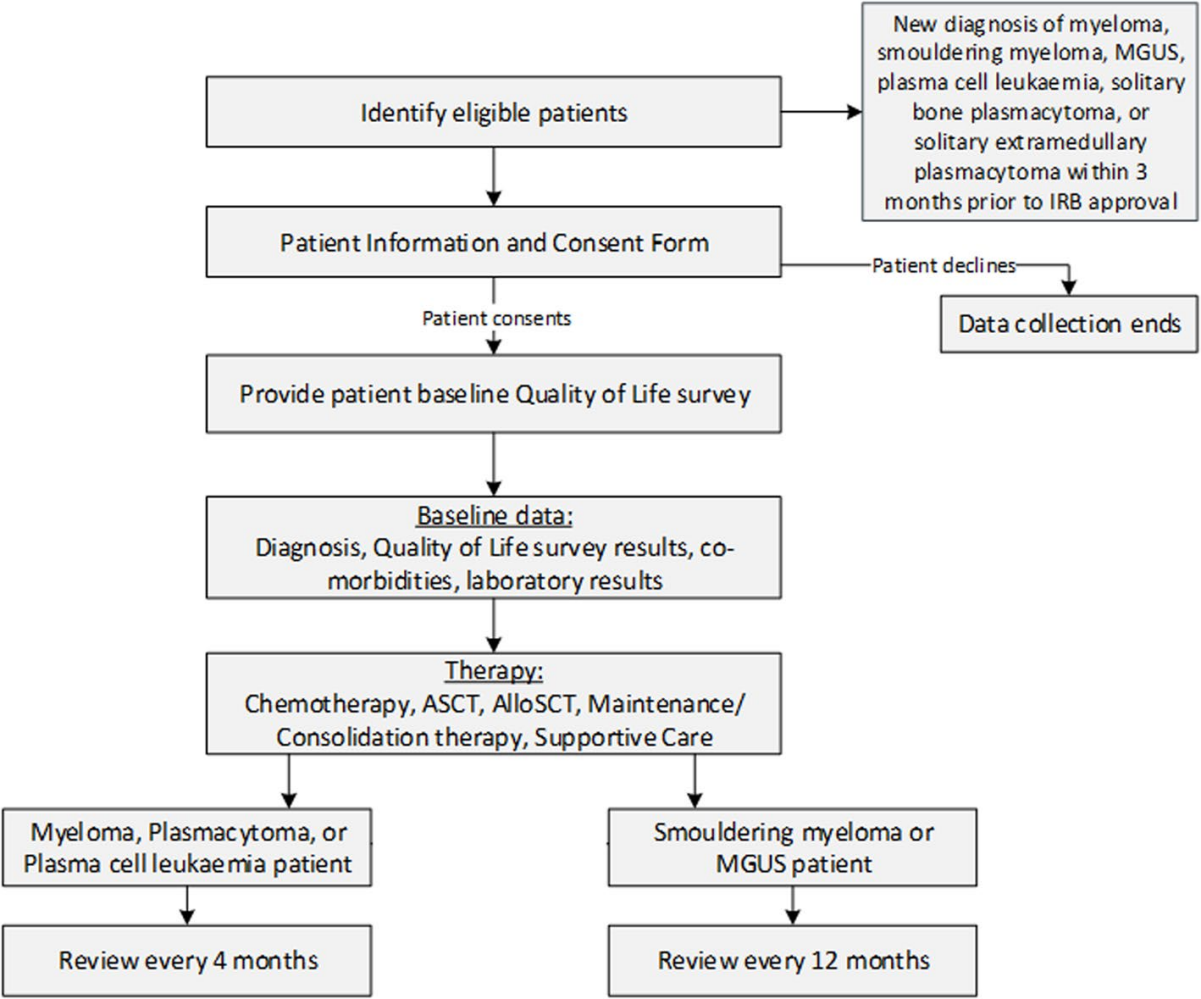
Key time points MGUS: monoclonal gammopathy of undetermined significance, IRB: international review board, ASCT: autologous stem cell transplant, AlloSCT: allogeneic stem cell transplant

Eligible patients are invited to participate by the Principal Investigators, or staff under their direction, at each hospital, and are followed throughout their treatment. Re-identifiable data on patient characteristics, diagnosis, medical history, treatment (including supportive therapies), and outcomes (response to treatment, overall and progression-free survival, and quality of life [using the EQ-5D-5L]) are entered to a bespoke SQL database by hospital staff and data managers (summarised in Fig. 2). Data are collected from hospital systems and the medical and pathology notes recorded during standard medical care from the time of diagnosis and throughout treatment. The data items collected, including the patient survey EQ-5D-5L, are standardised across the five APAC MRDR locations with the exception of Singapore where to comply with local privacy requirements, only year of birth is collected, and no postcode is collected. The frequency of patient review is diagnosis-dependent: four-monthly for patients diagnosed with MM or plasma cell leukaemia, and twelve-monthly for patients diagnosed with MGUS and smouldering MM (summarised in Fig. 3). Secure, region-specific databases and a corresponding web-based data entry portal with user-level permissions are used for data collection. Users are only permitted to view their own hospital data, and each database portal is geo-blocked and login protected. In addition, database portals are designed to log out after 20 min of inactivity to help prevent unauthorised use. Data and passwords are encrypted, and data are encrypted in transit and stored separately on secure servers managed by Monash University in Victoria, Australia. The hospitals remain the sole owners of their patient medical records. Intellectual property and ownership of the combined registry data rests with Monash University.

### Patient privacy considerations

In the planning phase, we consulted potential participating sites and investigators, HREC and IRBs, legal counsel, Monash University Information Technology services, and data privacy officers to assist with challenges presented by the different data privacy requirements and ethical regulations across the APAC region. Some modifications were made to the ANZ MRDR study design to accommodate the variation in local laws and regulations. For example, in Singapore, the hospital IRB did not permit collection of the participant’s postcode and date of birth – instead, year of birth is collected. In addition, some IRBs in multiple regions did not allow collection of the participant’s full name, to further protect patient privacy. To meet these requirements, before registration, each participant is allocated a patient code, which is stored securely at the participating hospital in a subject identification log with the participant’s name, date of birth, hospital identifier, and APAC MRDR registration number. This document is never shared with Monash University.

### Governance

Clinical experts in MM from the participating regions were identified through academic and industry contacts and invited to join the Steering Committee to provide local clinical context and oversight of the registry. The APAC MRDR is administered by the Study Coordinating Centre in the Transfusion Research Unit, School of Public Health and Preventive Medicine (SPHPM), Monash University, Melbourne, Australia. Over 40 clinical registries operate under the auspices of the SPHPM, including 10 managed by the Transfusion Research Unit, indicating a high level of experience and expertise in this area. Relevant staff members from the Transfusion Research Unit form part of the APAC MRDR Steering Committee to advise on the design and day-to-day operations of the registry.

### Funding

The APAC MRDR is an investigator-initiated study currently supported by Janssen-Cilag, producers of MM treatments daratumumab and bortezomib which are currently approved for use in much of the APAC region, including China more recently.

Janssen-Cilag does not influence the research activities of the registry but their funding support is acknowledged in publications and presentations. Industry funding permits a per-patient payment to participating hospitals for data entry and other registry-based operational activities.

### Hospital reporting

Six-monthly hospital reports of aggregate, age-adjusted outcome data at hospital- and region-level, are provided to contributing hospitals comparing patient characteristics, treatment, response to treatment and outcomes at their hospital to the rest of their jurisdiction, and to other areas in the Asia–Pacific region. These reports can be used to monitor and evaluate local practice and identify areas for improvement or learning opportunities for hospital staff, investigators and medical students. Additional analyses can be requested by hospitals, and participating hospitals can also download their own raw data at any time to conduct internal analyses for quality improvement purposes to evaluate patient characteristics, management and outcomes.

### Statistical analysis

Data were collected from commencement of recruitment in October 2018 to 18 March 2024; they are summarised as proportions, or median (interquartile range [IQR]) as appropriate. We calculated survival using Kaplan–Meier methods.

## Results

### Sites

**Fig. 4:**
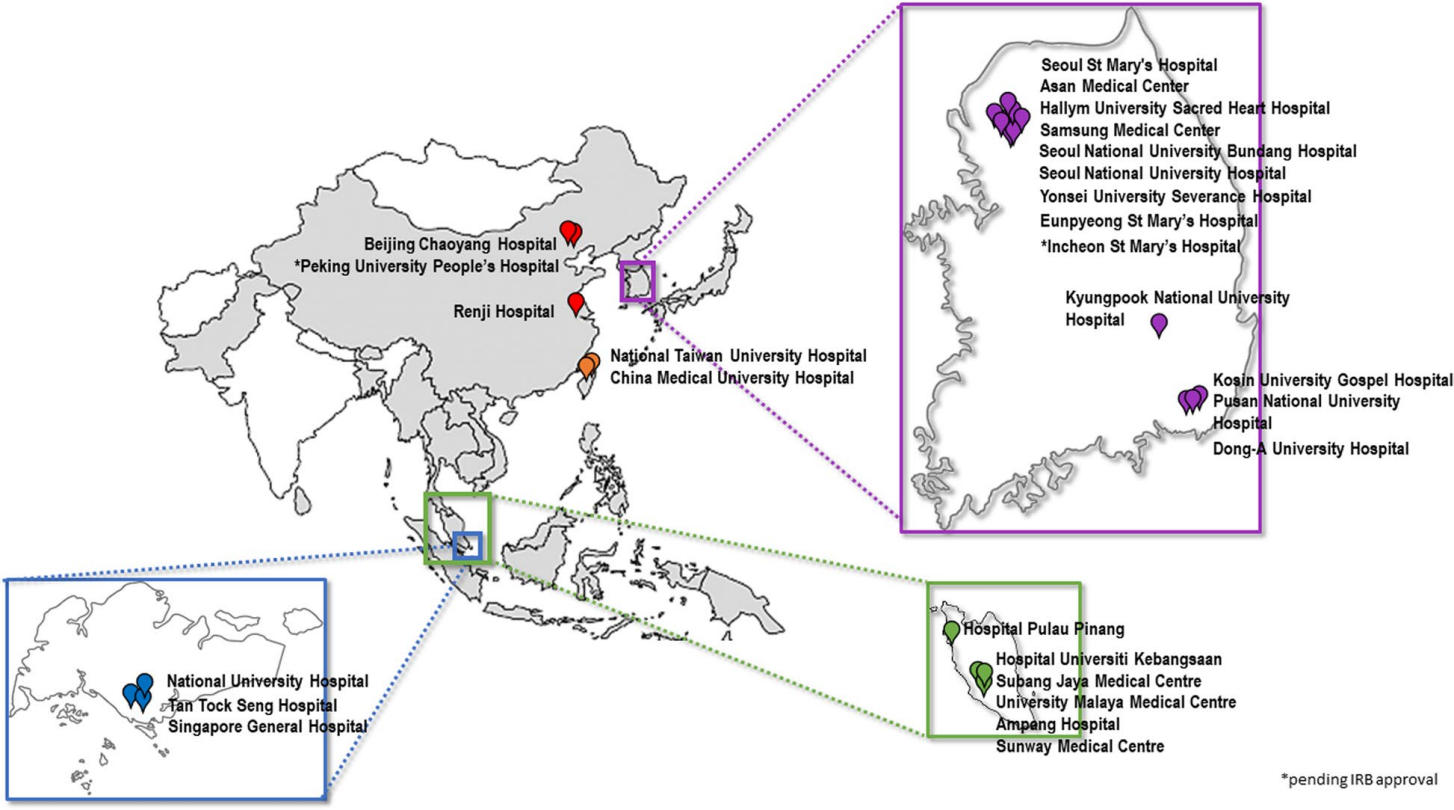
Participating APAC MRDR sites

By March 2024, 25 hospitals in Korea, Malaysia, Taiwan, Singapore, and China had received HREC or IRB approval to participate, and, of these, 23 hospitals had commenced patient recruitment (Fig. 4 and Table 1).

**Table 1:** Participating hospitals and patients registered to March 2024

	**Total**	**Korea**	**Singapore**	**Malaysia**	**Taiwan**	**China**
Hospitals with HREC/IRB^a^ approval	25	12	3	6	2	2
Active^b^ hospitals	23	11	3	6	2	2
Patients registered	1733	1344	197	154	35	3

^a^HREC/IRB: Human Research Ethics Committee or Institutional Review Board

^b^Active: hospitals with at least one patient registered

### Patients

As of March 2024, 1733 patients had been enrolled from Korea, Malaysia, Singapore, Taiwan and China. Table 1 shows the distribution of patient recruitment. At the time of analysis, diagnosis data were available for 1513 patients, of whom 86% (*n* = 1301) were newly diagnosed with MM, 3% (*n* = 46) with smouldering MM, 9% (*n* = 131) with MGUS, and 2% (*n* = 35) with other related diseases (solitary bone or extramedullary plasmacytoma or plasma cell leukaemia).

Table 2 shows preliminary baseline diagnostics (including high-risk cytogenetics) and demographic characteristics of newly diagnosed MM patients in Korea, Singapore, Malaysia and Taiwan.

**Table 2:** Newly diagnosed MM: baseline characteristics

	**Korea** *N* = 969	**Singapore** *N* = 159	**Malaysia** *N* = 141	**Taiwan** *N* = 32
Age, years, median (IQR)	65 (58, 72)	67 (59, 72)	63 (56, 69)	67 (61, 73)
Follow-up, months, median (IQR)	19 (6—36)	26 (11—43)	11 (6—18)	N/A
Gender, male	547/969 (56%)	91/159 (57%)	84/141 (60%)	21/32 (66%)
EQ-5D VAS score*, median (IQR)	70 (50, 80)	70 (65, 85)	78 (60, 90)	73 (60, 80)
ECOG performance status ≥ 2	157/946 (17%)	21/132 (16%)	58/138 (42%)	9/30 (30%)
Comorbidity present	398/957 (42%)	122/157 (78%)	75/140 (54%)	5/32 (16%)
Paraprotein type				
IgG	508/960 (53%)	94/158 (59%)	85/140 (61%)	20/32 (63%)
IgA	200/960 (21%)	32/158 (20%)	19/140 (14%)	3/32 (9.4%)
IgM	6/960 (.63%)	1/158 (.63%)	0/140 (0%)	0/32 (0%)
IgD	24/960 (2.5%)	2/158 (1.3%)	2/140 (1.4%)	0/32 (0%)
Light Chain Only Kappa	109/960 (11%)	16/158 (10%)	19/140 (14%)	3/32 (9.4%)
Light Chain Only Lambda	97/960 (10%)	13/158 (8.2%)	12/140 (8.6%)	6/32 (19%)
Non-Secretory MM	7/960 (.73%)	0/158 (0%)	3/140 (2.1%)	0/32 (0%)
Biclonal	9/960 (.94%)	0/158 (0%)	0/140 (0%)	0/32 (0%)
Cytogenetic high risk**	212/650 (33%)	31/128 (24%)	4/44 (9.1%)	0/8 (0%)

N/A: insufficient data; *Median EQ-5D Visual Analogue Scale Health State score at diagnosis (self-reported: 100 = best health imaginable, 0 = the worst); ECOG: Eastern Cooperative Oncology Group; **positive to either t(4:14), t(14:16) or Del17p by FISH

### Treatment

Table 3 shows the most frequently used treatment regimens and outcomes in the four jurisdictions. Regimens with bortezomib combined with an immunomodulatory drug were most frequently used in first-line therapy in all locations, with an overall response rate of > 90%; and lenalidomide-containing regimens were most frequently used in second-line therapy, with overall response rate > 70%. Progression-free survival median is shown for all patients, regardless of the treatment regimen received.

**Table 3:** Newly diagnosed MM: treatment and outcomes

	**Korea**	**Singapore**	**Malaysia**	**Taiwan**
Most frequently used first-line regimen	VTd 395/926 (43%)	VRd 46/156 (29%)	VTd 64/137 (47%)	VRd 11/27 (41%)
ORR* to first-line therapy	776/823 (94.3%)	120/129 (93.0%)	71/78 (91%)	18/19 (95%)
Most frequently used second-line regimen	KRd 83/241 (34%)	VRd 9/76 (12%)	VRd 9/32 (28%)	N/A
ORR* to second-line therapy	143/188 (76%)	42/56 (75%)	13/17 (76%)	N/A
Received ASCT†	331/586 (56%)	51/117 (44%)	27/68 (40%)	N/A
PFS, months, median (IQR)	33 (16-NR)	33 (-16-NR)	28 (17–34)	N/A

^*^ORR: overall response rate—proportion achieving partial response or better; †ASCT: only patients with at least 1-year post-diagnosis and with some follow-up data post-registration were included to allow time to have received a transplant; PFS: progression-free survival for all patients; N/A: insufficient data. V: bortezomib, T: thalidomide, d: dexamethasone, R: lenalidomide, K: carfilzomib, D: daratumumab

### Communication and site support

The APAC MRDR communicates regularly with participating sites, stakeholders, supporters and collaborators through several forums. Quarterly newsletters are circulated to interested parties, and a monthly email update is sent to participating sites. The APAC MRDR hosts an annual Investigators’ Meeting which is open for anyone to attend, including clinicians, site staff, researchers, students, patients and community organisations, and industry. At these meetings an overview of the registry, progress and current research are shared and attendees are encouraged to participate in discussion and to submit research proposals.

Support is available to participating site staff through an initial training session, including a tutorial on the APAC MRDR database; sites are also provided with supporting documentation including a database user guide and quick reference guide. Since 2022, annual Study Coordinator Meetings have been implemented to provide APAC MRDR data entry and research staff at sites with updates to the registry, including any changes to the database. This forum provides an opportunity to meet and learn from research staff working on the registry at other sites, and to discuss any difficulties encountered, challenging cases and areas that need clarifying.

Statistical support from qualified Monash staff is available to assist in the analysis of APAC MRDR data. If patient-level data are required, access to de-identified data is provided via a secure virtual environment equipped with statistical analysis software including Stata, SPSS and R.

## Discussion

The APAC MRDR is now established in Korea, Malaysia, Singapore and Taiwan, with China recently beginning patient recruitment. New information on the epidemiology of myeloma and related diseases, along with clinical practice and patient outcomes from sites across the region is becoming available, including for the first-time regional data on patient characteristics, therapeutic approaches and clinical outcomes.

### Challenges

The establishment of a myeloma registry in the APAC region has been an enormous and complex undertaking. Two major challenges have been the COVID-19 pandemic and changes to data privacy laws at participating locations, both of which have delayed the initial progress and expansion of the registry.

The COVID-19 pandemic had a substantial impact on myeloma patient management, hospital resources, and subsequently, clinical research activities [25, 26]. For many hospitals participating in the APAC MRDR, patient recruitment and data entry efforts ceased or vastly decreased and for many, hospital financial and staff resources were diverted away from research to hospital and national COVID-19 management activities. Operational changes to the APAC MRDR were implemented during this time. EQ-5D surveys and patient follow-up were often completed over the phone by the study coordinators, all study-related meetings were held via videoconference, and variables related to COVID-19 vaccination were added to the APAC MRDR databases. With the reduction in COVID-19 cases, some of the major metropolitan hospitals are now re-allocating resources to resume APAC MRDR data entry that was deferred during the peak of the pandemic. Despite these challenges, the APAC MRDR has continued to grow in the number of participants recruited and site activations.

Changes to data protection legislation have also presented challenges, leading to delays in the project to ensure compliance. For example, in November 2021, China’s Personal Information Protection Law, similar to the European Union’s General Data Protection Regulation (GDPR), came into effect. In addition, the Administrative Regulations on Human Genetic Resources (HGR) came into effect in July 2019; however, draft Implementation Rules on the Administrative Regulations on Human Genetic Resources were only released in March 2022 and came into effect on 1 July 2023. We required clarification from the ‘Implementation Rules’ on how HGR data is defined and whether data collected for the APAC MRDR is excluded or not from HGR notification. Navigating the different requirements, and how they apply to clinical registries, across different countries, has added complexity to the project.

### Future considerations

Future considerations for the registry include the consent model utilised and the collection of select patient identifiers. Currently, to satisfy local data privacy legislations, written patient consent is obtained before the patient is entered onto the database and most patient identifiers are not collected. However, for clinical registries such as the APAC MRDR, using an ’opt-out’ approach as described by Bergin and colleagues, and collecting select patient identifiers may help maximise patient participation, and facilitate audits and linkage with other datasets [13, 27]. We will monitor changes to privacy legislation that permit this approach to consent and the collection and release of patient identifiers for clinical research purposes in participating APAC MRDR locations as the registry progresses. In the meantime, audits or linkage with hospital databases and national cancer registries in the region will be explored to determine the extent of ascertainment bias.

### Strengths and limitations

We acknowledge the limitations of our study. Not all sites in each country are participating, and there is a higher proportion of participating hospitals and registered patients from South Korea, therefore, APAC MRDR patients therefore do not represent a typical cross-section of Asia–Pacific patients, due to differences in patient representation both between and within individual regions. Future analyses will be performed with this in mind, in order to mitigate the effects of this bias. The use of real-world data typically results in missing data in a number of fields, in part due to differences in practice (for example, some tests may not be performed due to restrictions on access to funding or diagnostic tools) or due to results not being entered even if they are available. We hold initial site training and regular data manager meetings to minimise the effects, and support is available at all times from the registry team at Monash. To provide an indication of data completeness, we provide fractions and denominators in all tables. This work also has a number of important strengths: the APAC MRDR is now a well-established, multiregional registry with a standardised MM dataset and international collaboration with clinical leaders in myeloma. The APAC MRDR has been designed for flexible, long-term data collection where new regimens and treatments can be easily incorporated, and patient management and outcomes can be monitored over time and compared between hospitals, regions and ethnicities**.** It is aligned with, and draws on, the network, infrastructure and strengths of the ANZ MRDR, which has now been operational for more than 10 years and is providing vital ‘real world’ data to government policy-makers, clinicians, patient groups and industry in Australia and New Zealand.

### Future directions

The APAC MRDR is maturing, and the collection of long-term patient follow-up data is a priority for the registry. Future plans include expanding to other APAC jurisdictions and developing location-specific working groups to encourage intra- and inter-region data analyses and research. The APAC MRDR is also collaborating with the ANZ MRDR on multiregional analyses and will seek to collaborate with other local registries and organisations on specific clinical and research questions. Although the registry is still maturing, APAC MRDR data have already been used in several investigator-initiated study proposals and presented at international scientific meetings [28–30]. Participating investigators are regularly encouraged via the APAC MRDR newsletter and investigator meetings to submit proposals. Linkage with local hospital, cancer, and death registries will be investigated for feasibility in the future to determine case ascertainment, and enhance the accuracy of survival data.

## Conclusions

The APAC MRDR is now established and expanding to include additional hospitals and locations in the region. As data mature, the registry will become an increasingly valuable resource for hospitals, clinicians, industry, and governments to provide real-world data on current myeloma treatment strategies and patient outcomes in the APAC region. Future plans include linkage with local cancer and health databases, development of a regional infrastructure to identify eligible patients for clinical trials and other research, and the provision of baseline diagnosis and treatment data for such studies.

## Data Availability

Access to data that support the findings of this study are available from the Asia–Pacific Myeloma and Related Diseases Registry (APAC MRDR) with permission from the Steering Committee and in accordance with the APAC MRDR Data Access and Publication Policy. More information is available at apacmrdr.org or via email to: sphpm.apacmrdr@monash.edu.

## Abbreviations

APAC: Asia-Pacific
APAC MRDR: Asia-Pacific Myeloma and Related Diseases Registry
ANZ MRDR: Australian and New Zealand Myeloma and Related Diseases Registry
GDPR: General Data Protection Regulation
HGR: Human Genetic Resources
HREC: Human Research Ethics Committee
IRB: Institutional Review Board
MGUS: Monoclonal gammopathy of undetermined significance
MM: Multiple myeloma
PCL: Plasma cell leukaemia
SPHPM: School of Public Health and Preventive Medicine
